# Integration of DNA Copy Number Alterations and Transcriptional Expression Analysis in Human Gastric Cancer

**DOI:** 10.1371/journal.pone.0029824

**Published:** 2012-04-23

**Authors:** Biao Fan, Somkid Dachrut, Ho Coral, Siu Tsan Yuen, Kent Man Chu, Simon Law, Lianhai Zhang, Jiafu Ji, Suet Yi Leung, Xin Chen

**Affiliations:** 1 Department of Bioengineering and Therapeutic Sciences, University of California San Francisco, San Francisco, California, United States of America; 2 Department of Surgery, Beijing Cancer Hospital & Institute, Peking University School of Oncology, Key Laboratory of Carcinogenesis and Translational Research (Ministry of Education), Beijing, China; 3 Liver Fluke and Cholangiocarcinoma Research Center, Faculty of Medicine, Khon Kaen University, Khon Kaen, Thailand; 4 Department of Pathology, The University of Hong Kong, Queen Mary Hospital, Pokfulam, Hong Kong; 5 Department of Surgery; The University of Hong Kong, Queen Mary Hospital, Pokfulam, Hong Kong; Institut Jacques Monod, France

## Abstract

**Background:**

Genomic instability with frequent DNA copy number alterations is one of the key hallmarks of carcinogenesis. The chromosomal regions with frequent DNA copy number gain and loss in human gastric cancer are still poorly defined. It remains unknown how the DNA copy number variations contributes to the changes of gene expression profiles, especially on the global level.

**Principal Findings:**

We analyzed DNA copy number alterations in 64 human gastric cancer samples and 8 gastric cancer cell lines using bacterial artificial chromosome (BAC) arrays based comparative genomic hybridization (aCGH). Statistical analysis was applied to correlate previously published gene expression data obtained from cDNA microarrays with corresponding DNA copy number variation data to identify candidate oncogenes and tumor suppressor genes. We found that gastric cancer samples showed recurrent DNA copy number variations, including gains at 5p, 8q, 20p, 20q, and losses at 4q, 9p, 18q, 21q. The most frequent regions of amplification were 20q12 (7/72), 20q12–20q13.1 (12/72), 20q13.1–20q13.2 (11/72) and 20q13.2–20q13.3 (6/72). The most frequent deleted region was 9p21 (8/72). Correlating gene expression array data with aCGH identified 321 candidate oncogenes, which were overexpressed and showed frequent DNA copy number gains; and 12 candidate tumor suppressor genes which were down-regulated and showed frequent DNA copy number losses in human gastric cancers. Three networks of significantly expressed genes in gastric cancer samples were identified by ingenuity pathway analysis.

**Conclusions:**

This study provides insight into DNA copy number variations and their contribution to altered gene expression profiles during human gastric cancer development. It provides novel candidate driver oncogenes or tumor suppressor genes for human gastric cancer, useful pathway maps for the future understanding of the molecular pathogenesis of this malignancy, and the construction of new therapeutic targets**.**

## Introduction

Gastric cancer is one of the most common malignancies and the second most common cause of cancer related death worldwide [1]. The major type of gastric cancer is adenocarcinoma, which can be further categorized into intestinal type and diffuse type [2]. Intestinal-type lesions are frequently ulcerative and occur in the distal stomach. Diffuse-type lesions are associated with a worse prognosis than the intestinal type. Surgical treatment is the only therapeutic modality that has a potentially curative effect to gastric cancer. The prognosis of gastric cancer patients depends heavily on the clinical and pathological stage of the disease at diagnosis. The 5-year survival rates after curative surgical resection decline from 60–90% in stage I to only 10–25% for patients in stage III of the disease [3]. Most gastric cancer patients are identified at the advanced stage, which leads to the dismal prognosis.

Genetic alterations are key events in the development of most tumors, including gastric cancer [4]. Studies suggest that tumor progression depends on the successive acquisition of chromosomal aberrations leading to gains or losses of part of the tumor cell genome. Therefore, characterization of genomic abnormalities may help elucidate the molecular pathogenesis of gastric cancer as well as reveal the genetic markers of progression. Array-based comparative genomic hybridization (aCGH) is a powerful method used to identify pathogenic DNA copy number changes on a genome-wide scale [5]. aCGH has been applied to a number of solid tumors, including gastric cancer [6], [7]. It has been shown to be useful in the identification of novel oncogenes and tumor suppressor genes, and to classify tumors based on genetic changes.

Expression profiling experiments identified a large numbers of genes which are differentially expressed in normal and tumor tissues. However, most of these genes are likely to be passenger genes which have limited contribution to tumorigenesis. The key challenge has been to identify driver oncogenes or tumor suppressor genes that play important roles during tumor initiation and progression, Genomic DNA copy number variation is an important type of genetic alteration observed in tumor cells, and it contributes to tumor evolution by alterations of the expression of genes within the region [8]. DNA copy number gains and losses are not random, but rather represent consistent genetic events during carcinogenesis. Identification of genes that are both over-expressed and amplified or under-expressed and deleted may be beneficial because these genes may represent driver genetic alterations.

Previous studies have reported DNA copy number changes or expression profiles in gastric cancer samples. The studies have also identified common chromosome gains and losses, as well as hundreds of genes that may distinguish tumors from normal tissues [6], [9]. However, few studies have investigated the association between DNA copy number variations and transcriptional expression profiles. In this manuscript, we performed aCGH analysis in a large number of human gastric cancer samples. Furthermore, integrated analysis of DNA copy number variations and corresponding gene expression values was performed to identify significant genes that may contribute to gastric cancer pathophysiology. A total of 321 candidate oncogenes and 12 candidate tumor suppressor genes were identified through the analysis.

## Materials and Methods

### Ethic Statement

The use of archival gastric specimen for the current study was approved by the Ethics Committee of the University of Hong Kong and the Internal Review Boards of University of California, San Francisco.

### Tumor Samples, Cell Lines and DNA, RNA Preparation

Tumor samples were collected from gastrectomy specimens from the Department of Surgery, Queen Mary Hospital, The University of Hong Kong. Eight gastric cancer cell lines AGS, BGC823, N87, NUGC3, SNU16, SNU5, KATOIII and MNK45 have been described in our previous publications [10]. Genomic DNA was extracted using the Genomic DNA purification Kit (Qiagen, Valencia, CA).

The clinico-pathological parameters of the tumors have been previously published [11]. Tumors were classified using Lauren’s classification of intestinal, diffuse, mixed, and indeterminate types [2]. The presence of H. pylori in the gastrectomy specimens was determined by histological examination and supplemented by modified Giemsa staining. The presence of EBV in cancer cells was assayed by in situ hybridization for EBER as previously described [12]. The tumor stages were defined by the General Rules for Gastric Cancer Study of the Japanese Research Society for Gastric Cancer [13].

### Array-based CGH

Human 1.14 arrays were obtained from the UCSF Cancer Center Array Core (http://cc.ucsf.edu/microarray/). They consisted of 2353 bacterial artificial chromosome (BAC) clones that covered the human genome at 1.5 Mb resolution. For hybridization, 1 µg of tumor DNA and 1 µg of gender matched reference DNA was labeled by random priming using Cy3-dCTP and Cy5-dCTP, respectively, with the Bioprime Kit (Invitrogen). Unincorporated fluorescent nucleotides were removed using a Sephadex G-50 column (Amersham, Piscataway, NJ). Sample and reference DNA were mixed with 100 µg Cot-1, precipitated, and resuspended in hybridization solution. The hybridization solution was denatured for 10 min at 72°C and then incubated for 1 h at 37°C. Hybridization was performed for 48–72 hrs in a moist chamber on a slow rocking table. Arrays were washed for 10 min in 50% formamide and 2×SSC at 45°C, and 10 min in phosphate buffer at room temperature. Slides were mounted in mounting solutions containing 0.3 µg/ml DAPI. Three single-color intensity images (DAPI, Cy3 and Cy5) were collected for each array using a charge coupled device camera.

### Array-based CGH Data Analysis

The UCSF SPOT software [14] was used to automatically segment the spots based on the DAPI images, perform local background corrections, and calculate various measurement parameters including log2 ratios of the total integrated Cy3 and Cy5 intensities for each spot. Raw data of the aCGH are available at GEO (accession number: GSE33501).

Program SPROC was used to associate clone identities and a mapping information file with each spot so that the data could be plotted relative to the position of the BACs. Chromosomal aberrations were classified as a gain when the normalized log2 Cy3/Cy5 ratio was >0.225 and as a loss when the ratio was <−0.225. The number was determined as 3-fold the average SD of normal versus normal aCGH hybridization. Amplifications were identified when the normalized log2 Cy3/Cy5 ratio was >0.8. Similarly, homozygous deletions were identified when the normalized log2 Cy3/Cy5 ratio was <−0.7. Multiple gains, losses, and amplifications were counted as separate events. The threshold of gain or loss of an entire chromosome arm was defined as the median log2 ratio of >0.225 or <−0.225 for all clones on the chromosome arm.

### Statistical Data Analysis

Samples were categorized based on the experimental results and compared with the clinical data (Table S1) using significant analysis of microarray (SAM) analysis [15]. DNA copy number alterations including median percentage of gain and loss. Frequent amplification and deletion were analyzed by using CGH explorer 3.2 (http://www.ifi.uio.no/forskning/grupper/bioinf/Papers/CGH/). “Analysis of Copy Errors” (ACE) was performed using a false discovery rate (FDR) of 0.0001 and medium sensitivity. Clustering of all samples was performed in TreeView version 1.60.

R/Bioconductor software, including the CBS program, was used to compute the correlation between copy number change and gene expression. The expression data of the 6688 cDNA clones used in the previous gene expression analysis [11], [16] (GEO accession number: GSE2701) was retrieved. Mapping position for these cDNA clones were assigned using the NCBI genome assembly, accessed through the UCSC genome browser database (NCBI build 35). The aCGH data was segmented using circular binary segmentation (CBS) as implemented in the DNA copy package in R/Bioconductor to translate experimental intensity measurements into regions of equal copy numbers. Missing values for clones mapping within segmented regions of equal copy numbers were imputed by using the value of the corresponding segment. The gene expression clones were mapped to the BAC clone within 1 Mb of the gene expression clone which had the highest Pearson correlation between copy number and gene expression. “Smoothed” values from CBS with the originally observed log2 ratio for the outlier clones described above and the imputed values for missing values were considered in computing correlation with gene expression. Correlation was only computed for clones, and a correlation coefficient of 0.29 was used as the cut-off to identify clones having positive correlation between copy number and gene expression. *p*-values for the gene expression and copy number correlations were obtained based on permutation. The labels of expression data were randomly shuffled and the Pearson correlation between gene expression clones and copy number BAC clones were calculated as described previously. This was repeated 1000 times. For each gene expression clone, the p-value was determined as the proportion of times the permutation based correlation was greater than or equal to the observed correlation. The *p*-values were then corrected for multiple testings by controlling for the false discover rate (FDR) using the Benjamini-Hochberg method [17].

Functional analysis of the significant genes was performed using Ingenuity Pathway software (Ingenuity Systems, Redwood City, CA).

## Results

### Array-based CGH Analysis of Human Gastric Cancer

**Figure 1:**
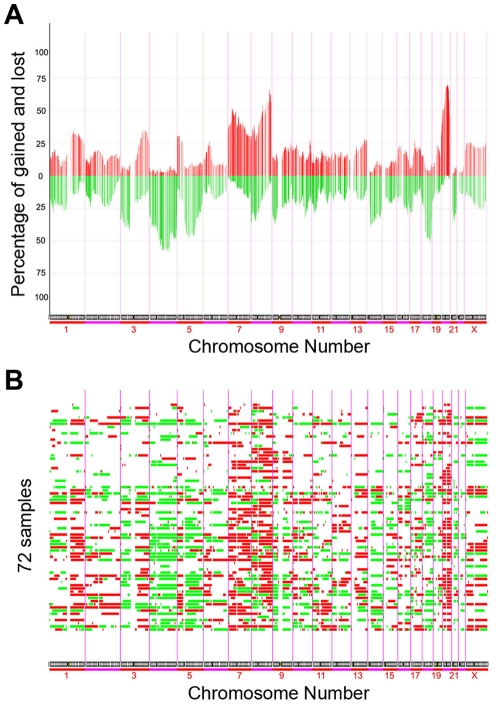
DNA copy number alterations by aCGH. (A) Overall frequency of DNA copy number alterations by aCGH. Frequency analysis measured as a fraction of cases gained or lost over all the BAC clones on the arrays. Data presented was ordered by chromosomal map position of the clones. Lower bars represented losses and upper bars represented gains. The purple vertical bars represented the boundary between each chromosome. (B) DNA copy number alterations in each gastric cancer samples. 72 tumor samples were ordered from top to bottom. Red columns represented copy number gains and green columns represented copy number losses.

To identify DNA copy number alterations in gastric cancers, we applied BAC aCGH to 64 human gastric cancer tissue samples and 8 gastric cancer cell lines. The raw data are available in Table S2. We observed recurrent chromosomal variations in these samples, and regions with significant DNA copy number changes were identified. The resulting frequency plot and aberration plot of gains and losses are shown in Figure 1A and Figure 1B respectively. Two representative genome-wide ratio plots for individual gastric tumor are shown in Figure S1. The most common DNA copy number variations in this set of human gastric tumors as determined by the median percentage of gain or loss included gains of 5p, 8q, 20p, 20q, and losses of 4q, 9p, 18q, 21q.

Next, we analyzed DNA copy number variations in gastric cancer samples with different clinico-pathological features including tumor stage, tumor type, tumor site, tumor differentiation, Helicobacter pylori and EBV infection, as well as the difference between gastric tumor samples and cell lines, (Figure S2, S3, S4, S5, S6, S7, S8 and Table S3). We found specific chromosomal aberrations enriched in certain clinico-pathological features. For example, loss of 19p was more frequently observed in stage 1 & stage 2 tumors (20%) than in stage 3 & stage 4 samples (3.41%) (Table S3). 16p loss was identified in 10% of the Helicobacter pylori negative samples compared with 0% in the Helicobacter positive samples, while 16p gain was observed in 14.71% of the Helicobacter pylori positive samples but only in 3.33% of the Helicobacter negative samples (Table S3). These results suggest the possible contribution of genes within specific regions to specific tumor phenotypes.

**Figure 2:**
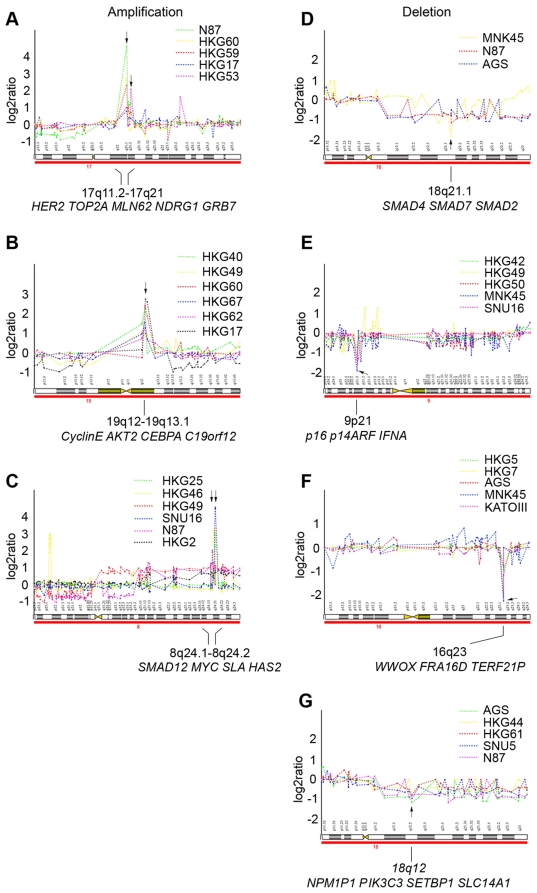
DNA copy number analysis of representative amplicons and homozygous deletions. Clones were ordered by their position from pter (left) to qter (right). The log2 ratios of every clone in these specific cases were plotted as broken line graphs with different color. Multiple clear copy number changes (gains, losses, amplifications and deletions) can be recognized. The center of amplicon and homozygous deletion cores were indicated together with genes in each core region. (**A**) Amplification in 17q11.2–17q21. (**B**) Amplification in 19q12–19q13.1. (**C**) Amplification in 8q24.1–8q24.2. (**D**) Homozygous deletion in 18q21.1. (**E**) Homozygous deletion in 9p21. (**F**) Homozygous deletion in 16q23. (**G**) Homozygous deletion in 18q12.

High-level amplifications and homozygous deletions are summarized in Table S4. The most frequent amplification was found at the long arm of chromosome 20. In this region, four separate focal amplicons could be identified: 20q12 (7/72), 20q12–20q13.1 (12/72), 20q13.1–20q13.2 (11/72) and 20q13.2–20q13.3 (6/72). The second most frequent amplification, occurring in the long arm of chromosome 8, also had four separate focal amplicons: 8q23.1 (3/72), 8q24.1 (7/72), 8q24.12–8q24.2 (6/72) and 8q24.2 (6/72). The most frequent homozygous deletion region was found at 9p21 (8/72) and at 18q22 (6/72). Other high-level amplifications and homozygous deletions occurred at relatively lower frequencies. Examples of frequent aberrations are shown in Figure 2. Some well-characterized oncogenes (e.g., *HER2, TOP2A, CyclinE, TGFB1, AKT2, MYC*) and tumor suppressor genes (e.g., *P16, SMAD4, SMAD7*) are found to be located in these loci. Interestingly, a higher number of amplifications and homozygous deletions were identified in the cell lines than in the primary gastric cancer tumor samples.

### Contribution of Genomic DNA Copy Number Variation to Global Gene Expression Changes in Human Gastric Cancer Samples

In our previous study, we reported the gene expression profile in 90 primary gastric cancer samples compared with their 14 metastatic counterparts and 22 non-neoplastic gastric mucosae, with 6688 cDNA clones showing significant variation across these samples [11], [16]. Among the 90 gastric cancer samples, 62 specimens were included in the current aCGH study. In order to determine whether genomic DNA copy number variations contribute to global gene expression pattern changes, we determined the correlation between gene expression values and the corresponding DNA copy number changes in these 62 human gastric cancer samples on a gene by gene basis. Of the 6688 cDNA in the original expression studies, 5719 cDNA clones with position information were retrieved for this analysis. Of these 5719 cDNA clones, 1352 cDNA clones (23.6% of the total cDNA clones analyzed), representing approximately 973 unique genes, showed statistical significant correlation between expression values and DNA copy number variations (correlation >0.29 and adjusted p value less than 0.01 with FDR less than 3.4%. See Table S5 for the list of genes). To illustrate whether DNA copy numbers influence gene expression, we compared the pair wise correlation of gene expression data with either aCGH values of BAC clones close to the locus where each gene is located at (diagonal), or aCGH values of BAC clones located at other regions of the genome. We found pairs of regions along the diagonal have higher positive correlation (median correlation ∼0.12) than the off-diagonal pairs (median correlation ∼0.0) (Figure S9A). A heatmap of the pairwise correlation between gene expression and copy number also demonstrates the positive correlation along the diagonal (Figure S9B).

Overall, our data confirm that genomic DNA copy number variations contribute to the regulation of regional gene expression profiles in human gastric cancer samples.

### Identification of Candidate Oncogene or Tumor Suppressor Genes for Human Gastric Cancers

To pinpoint candidate oncogenes or tumor suppressor genes, we applied two criteria to the list of 1352 cDNA clones which showed statistically significant correlation between gene expression and corresponding DNA copy number changes. First, we searched for genes that showed 5 more gains than losses or 5 more losses than gains in gastric cancer samples. Next, we matched the gene list with the 3329 cDNA clones that were identified to be differentially expressed between non-tumor gastric tissues and human gastric cancer samples [11]. Thus, we narrowed our list to 363 clones, representing 333 unique genes (Table S6). Among these genes, 321 genes were up-regulated in gastric cancer samples and were frequently gained or amplified at the genomic DNA level. The remaining 12 genes were down-regulated in gastric cancer samples and were frequently deleted at the genomic DNA level. These two set of genes, therefore, represent potential candidate oncogenes or tumor suppressor genes, respectively, which may be involved in gastric cancer pathogenesis and development.

### DNA Copy Number Changes with the Corresponding Gene Expression Values in Selected Gene Clusters in Human Gastric Cancers

**Figure 3:**
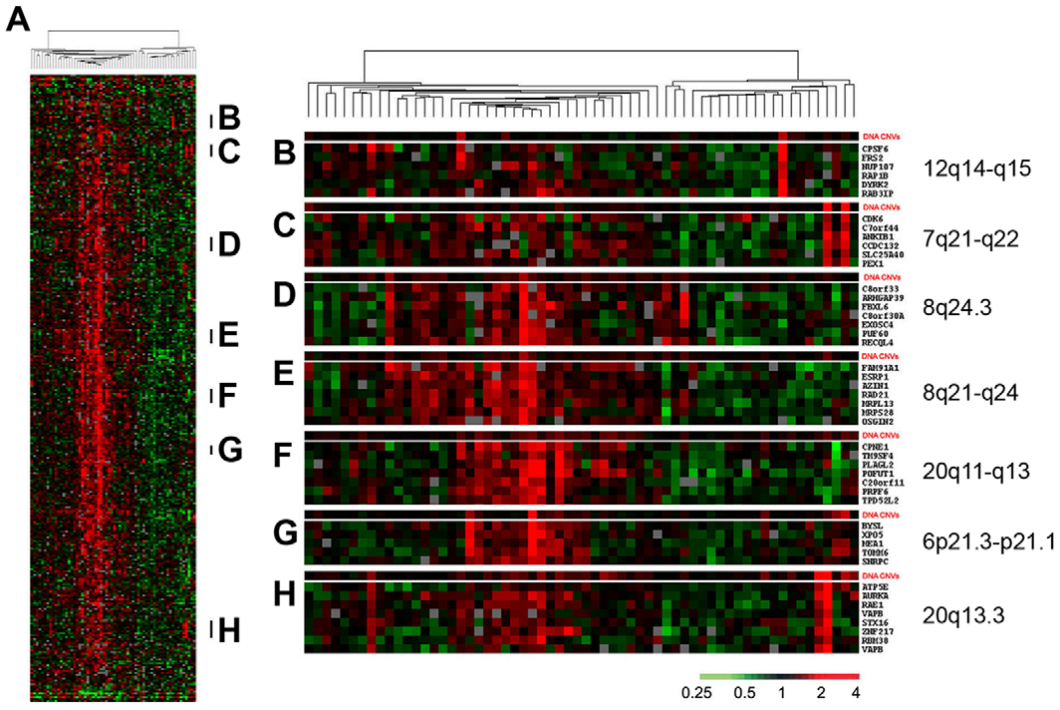
Hierarchical clustering of gastric tumors. (**A**) Hierarchical clustering the patterns of variation in expression of 333 candidate oncogene and tumor suppressor genes (from Table S6) in 62 gastric tumors**.** Each row represented a separate cDNA clone on the microarray and each column represented the expression pattern in a separate tumor or tissue sample. The ratio of abundance of transcripts of each gene to its mean abundance across all tissue samples was depicted according to the color scale shown at the bottom. Gray indicated missing or excluded data. The dendrogram at the top of the figure represented the hierarchical clustering of the tumors based on similarity in their pattern of expression of these genes. (**B**) **to** (**H**) compared DNA copy number changes with the corresponding gene expression values in selected gene clusters in each individual tumor sample. See Table S8 for full data.

To further illustrate how DNA copy number variations influence gene expression, we analyzed the expression patterns of the 333 candidate oncogenes and tumor suppressor genes in the 62 gastric cancer samples using hierarchical clustering (Figure 3A). No associations were identified between the clustering pattern and clinical features (Figure S10), suggesting that these genes do not provide additional values for molecular classification of human gastric cancer. Interestingly, several gene clusters were found to be located at the same chromosomal regions, including genes located at 6p21.3–p21.1, 7q21–q22, 8q21–q24, 8q24.3, 12q14–q15, 20q11–q13 and 20q13.3 (Figure 3B to 3H). An overall strong correlation between coordinated upregulated expression of these gene clusters and DNA copy number gains in the corresponding chromosomal regions was observed (Figure 3B to 3H). It suggests that DNA copy number variation is a key contributor to the expression variation of these genes within the cluster.

### Pathway Analysis of Significantly Expressed Genes

**Figure 4:**
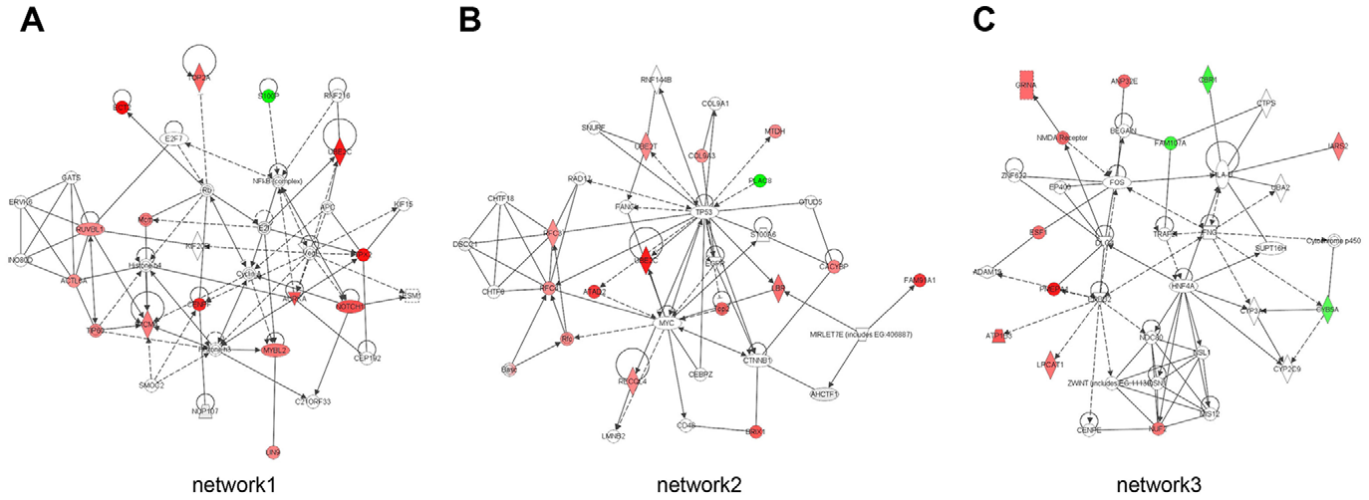
Ingenuity networks in gastric cancer samples. Ingenuity networks generated by mapping the candidate oncogenes and tumor suppressor genes identified by integrated analysis of expression array and aCGH data. Each network was graphically displayed with genes or gene products as nodes (different shapes represented the functional classes of the gene products) and the biological relationships between the nodes as edges (lines). The length of an edge reflected the evidence in the literature supporting that node-to-node relationship. The intensity of the node color indicated the degree of up- (red) or downregulation (green) of the respective gene. Genes in uncolored notes were not identified as differentially expressed in our experiment and were integrated into the computationally generated networks on the basis of the evidence stored in the IPA knowledge memory indicating a relevance to this network. A solid line without arrow indicated protein-protein interaction. Arrows indicated the direction of action (either with or without binding) of one gene to another.

The Ingenuity Pathway Analysis (IPA) software was employed to investigate the interactions among the candidate oncogenes or tumor suppressor genes identified by expression array and aCGH. Figure 4 shows the three most significant networks of interaction in gastric cancer samples. Network 1 was specifically associated with cancer, renal & urological disease, and cell cycle. Network 2 was specifically associated with connective tissue development and function, cancer, and gastrointestinal disease. Network 3 was specifically associated with genetic disorder, skeletal & muscular disorders, and inflammatory disease (Table S7). All networks reached a score of 21 or higher and contained 11 or more genes, which demonstrated the extensive relationship and interaction among the significantly regulated genes in gastric cancer. Top biological functions of these genes were related to cell cycle, DNA replication, recombination and repair, energy production, and nucleic acid metabolism (Figure S11). All these functions are known to be involved in tumorigenesis, providing possible links between the identified candidate oncogenes and tumor suppressor genes during gastric cancer development.

## Discussion

Gene copy number alterations are particularly important as deregulating events in cancer progression. In this study, we analyzed Copy Number Aberrations (CNAs) by array CGH. Frequent gains and losses were identified from the study. Furthermore, chromosomal regions with high levels of amplifications and homozygous deletions were also described. Additionally, correlation between gene expression and DNA CNAs were investigated. Candidate oncogenes and tumor suppressor genes were identified by performing integrated analyses of genome copy number and gene expression. Finally, relationships among these candidate genes and their biological function were described in 3 networks using the Ingenuity pathway analysis. The data support that combining aCGH and gene expression array analysis is a powerful method to identify candidate oncogenes or tumor suppressor genes in human gastric cancer. Consistent with this paper, previous studies have applied similar approaches to identify driver genetic events in other tumor types, such as liver cancer [18] and breast cancer [19]. Interestingly, more candidate oncogenes were identified than candidate tumor suppressor genes in our study. It could be explained by the larger possible magnitude range of gain compared to loss in tumor samples combined with compressed ratios from admixed non-tumor cells. The difference in gene numbers may also suggest that the expression of oncogenes may be more profoundly regulated by CNAs than tumor suppressor genes are.

In the aCGH analysis, frequent gains and amplifications were detected in gastric cancer samples. Of note, consistent with previous studies [20], [21], 20q was the most frequent site of gain detected in gastric cancer samples. Amplification at 20q has also been reported in several other cancers, such as breast cancer [22] and pancreatic cancer [23]. In our study, high level amplifications were found at 20q12–q13.3 in gastric cancer. Several genes are located at this locus, such as *AIB1* and *BCAS1*. *AIB1* (20q12), a steroid receptor co-activator first found amplified in breast and ovarian cancer, is involved in gastric cancer cell proliferation through interaction with nuclear receptors [24]. *BCAS1* (20q13.2), breast carcinoma amplified sequence 1, is amplified in a variety of tumor types and is associated with more aggressive tumor phenotypes. Up-regulated expression of *BCAS1* is significantly correlated with the high level amplification of 20q13 in adenocarcinomas of the gastro-esophageal junction [25]. 8q was the second most frequent site of gain as it was detected in 26.39% of the samples. Amplification at 8q has been identified in many cancers, such as breast cancer and pancreatic cancer [23], [26]. In our study, high level amplifications were found at 8q24.1–q24.2 in gastric cancer. Several genes are located at this locus. *MYC* is the most representative one. It is one of the most studied oncogenes, which contributes to the malignancy of many different aggressive and undifferentiated human cancers [27]. The pathologic effect of *MYC* has been ascribed to its ability to control multiplecellular processes such as cell growth, differentiation, apoptosis, DNA damage response, genomic instability, angiogenesis, and tumor invasiveness [28].

Another important high level amplification was found at 17q12–q21. The representative genes located at this locus is *ERBB2*. Overexpression and/or amplification has been observed in many kinds of cancers, including gastric cancer [29], [30], [31]. Correlation between *ERBB2* amplification and overexpression is noted by comparing aCGH and expression array data in our gastric cancer data set (Figure S12). Overexpression and amplifications were identified in only a small number (∼6 of 72) of gastric cancer samples. This may explain why ERBB2 was not selected in the correlated candidate oncogene list as it did not pass the criteria as one of the differentially expressed genes. Nevertheless, the result clearly suggests that amplification of 17q12–q21 may represent a key mechanism for high levels of ERBB2 expression in a subset of human gastric cancer samples. Gastric cancer patients with 17q12–q21 amplification may benefit from treatment with Herceptin, a humanized antibody, designed to target and block the function of ERBB2.

Consistent with the study by *Gorringe KL, et al*, amplifications at 6p21 and 5p13 were also identified in our array CGH results [7]. It is intriguing to note that a disproportionally higher numbers of high-level amplifications and homozygous deletions were identified in gastric cancer cell lines compared to tissue samples. The observation indicates that these amplifications or deletions may provide growth advantages during *in vitro* cell culture, and therefore are enriched in cell lines. The results highlight the importance of these high-level amplifications and homozygous deletions in regulating cell proliferation or survival. The cell lines with these amplifications or deletions provide excellent resources to help further study the functional roles of the genes within these regions during gastric cancer development.

Previous studies have provided insights into the importance of specific copy number alterations in the development of epithelial tumors, showing that these alterations may lead to the altered expression of critical oncogenes or tumor suppressors [21], [31], [32]. Our study therefore confirms these previous reports and provides evidence to support that CNA represents an important factor in regulating the abnormal up or down-expression of these genes during gastric cancer carcinogenesis. However, most of the genes identified from our studies are still likely to be passenger genes whose expression are highly gene-dose dependent, and have limited functional roles during tumorigenesis. Since these CNVs are not random and the main consequence of CNVs in tumors cells is likely to be the de-regulation of the expression of genes important for tumorigenesis, driver oncogenes and tumor suppressor genes are likely to be included in the large number of genes that we have identified. Clearly, further functional analysis is required to identify these driver oncogenes and tumor suppressor gene among our genelist. To achieve this goal, one can apply a siRNA based screen to silence the expression of candidate oncogenes in gastric cancer cell lines. Similar studies have been performed using breast cancer cell lines. Such functional screens prove to be critical to narrow down the true driver oncogenes. For example, Thollet A *et al* showed that siRNA-mediated silencing of ZNF217 expression in MCF7 breast cancer cells led to decreased cell proliferation and increased sensitivity to paclitaxel [33].

Overall, our studies provide a list of candidate genes that need to be further investigated functionally. Nevertheless, the genelist already provides some interesting genes as candidate oncogenes whose oncogenic potential has been demonstrated in other tumor types. The genes include *NOTCH1* [34], [35], [36], *BMI1* [37], [38], [39], [40], [41], *EFNA1* [42], [43], *NCOA2* [44], *BYSL* [45], [46], and *RAD21* [47]. For example, Notch1, a member of Notch family receptor has been indicated as an oncogene in multiple tumor types. High expression of *NOTCH1* was observed in human breast cancer and colorectal cancer, both of which are correlated with poor outcome of cancer patients [34]. Activated *NOTCH1* induced lung adenomas in mice and cooperated with Myc in the generation of lung adenocarcinoma [35]. Recent studies showed that the Notch1 receptor intracellular domain (N1IC), the activated form of Notch1 receptor, was associated with gastric cancer progression through cyclooxygenase-2 [36]. Therefore, Notch signaling pathway may be a new target for treatment of gastric cancer. A second example is Bmi1. *BMI1*, B-cell-specific Moloney murine leukemia virus insertion site 1, is a member of a polycomb group of transcriptional repressors and was originally identified as an oncogene associated with c-myc in the development of murine lymphoma [37]. Additional work has revealed that BMI1 had been associated with tumor development and progression. For example, BMI1 alone has been shown to induce malignant transformation of HaCaT cells [38]. Up-regulation of BMI1 can promote cell proliferation and prevent apoptosis [39]. Moreover, BMI1 is related to proliferation, survival, and poor prognosis in pancreatic cancer [40]. Recently, high expression of BMI1 was observed both in gastric cancer cell lines and gastric tumors. Overexpression of BMI1 was found to be correlated with advanced clinical stage and lymph node metastasis in gastric cancer patients [41]. Taking it all together, BMI1 may become a new biomarker in supporting the diagnosis and determining the prognosis of gastric cancer in clinical practice though more studies still should be done.

Our study also identified several candidate tumor suppressor genes. *IQGAP2* is such a candidate. Several studies have already suggested the tumor suppressing activity of IQGAP2. For example, it was showed that *IQGAP2* deficiency results in an 86% incidence of hepatocellular carcinoma in *IQGAP2* knockout mouse model [48]. IQGAP2 expression is downregulated in more invasive and metastatic liver cancer cell lines as well as most human hepatocellular carcinoma tissue [49]. Additionally, *IQGAP2* inactivation by hypermethylation is found in human gastric cancer samples [50] and *IQGAP2* knockdown with siRNA increased the invasive capacity of MKN45 gastric cancer cell line. Our study showed that down-regulation of IQGAP2 is also regulated by DNA copy number loss. The discovery of both genetic and epigenetic mechanisms for repressing IQGAP2 expression in gastric cancer provides strong evidence in support of *IQGAP2* acting as a tumor suppressor gene and calls for further investigation on the role of IQGAP2 in gastric tumor development.

## Supporting Information

Figure S1 **Two representative genome-wide ratio plots for individual gastric tumor.** Log2 ratio for each of the genomic clones was plotted according to chromosome position. (A) Whole genome DNA copy number profile of gastric cancer tissue sample HKG24T. Note that this sample showed the following DNA copy number variations: +3q, +5p, +8q, +13q, +17p, −4q, −10p and −18q. (B) Whole genome DNA copy number profile of gastric cancer cell line N87. Note that this sample showed the following DNA copy number variations: +5p, +8q, +11q, +20q, −3p, −5q, −6p, −6q, −7q, −8p, −11p, −14q, −17p and −21q. In addition, it also has amplification at 8q21, 8q24, 11q22 and 17q21.
(PDF)

Figure S2 **DNA copy number variations in gastric cancer samples with different tumor differentiation.** Data presented are ordered by chromosomal map position of the clones. Lower green bars represent losses or deletions, and the upper red bars represent gains or amplifications. (A) Well & moderate differentiated tumor. (B) Poor differentiated tumor.
(PDF)

Figure S3 **DNA copy number variations in gastric cancer samples with different tumor site.** Data presented are ordered by chromosomal map position of the clones. Lower green bars represent losses or deletions, and the upper red bars represent gains or amplifications. (A) Tumor site: antrum. (B) Tumor site: body. (C) Tumor site: cardia.
(PDF)

Figure S4 **DNA copy number variations in gastric cancer samples with different tumor stage.** Data presented are ordered by chromosomal map position of the clones. Lower green bars represent losses or deletions, and the upper red bars represent gains or amplifications. (A) Stage 1 & 2 tumor. (B) Stage 3 & 4 tumor.
(PDF)

Figure S5 **DNA copy number variations in gastric cancer samples with different tumor type.** Data presented are ordered by chromosomal map position of the clones. Lower green bars represent losses or deletions, and the upper red bars represent gains or amplifications. (A) Tumor type: diffuse type. (B) Tumor type: intestinal type.
(PDF)

Figure S6 **DNA copy number variations in gastric cancer tumors or cell lines.** Data presented are ordered by chromosomal map position of the clones. Lower green bars represent losses or deletions, and the upper red bars represent gains or amplifications. (A) Tissue samples. (B) Cell lines.
(PDF)

Figure S7 **DNA copy number variations in helicobacter pylori negative or positive gastric cancer samples.** Data presented are ordered by chromosomal map position of the clones. Lower green bars represent losses or deletions, and the upper red bars represent gains or amplifications. (A) Helicobacter pylori negative. (B) Helicobacter pylori positive.
(PDF)

Figure S8 **DNA copy number variations in EB virus negative or positive gastric cancer samples.** Data presented are ordered by chromosomal map position of the clones. Lower green bars represent losses or deletions, and the upper red bars represent gains or amplifications. (A) EB virus negative. (B) EB virus positive.
(PDF)

Figure S9 **Correlation between DNA copy number variations and global gene expression patterns.** Each chromosomal arm was divided into equal number of parts or bins of size 20 Mb and then average pairwise Pearson correlation between gene expression and copy number was calculated for all pairs of binned regions. (A) Box plots of correlation between pairs along the diagonal (cDNA clones with surrounding BAC clones) and pairs off diagonal (cDNA clones with unrelated BAC clones). (B) Heatmap of the average correlation between gene expression and copy number.
(PDF)

Figure S10 **Correlations between the clustering pattern and clinical features of gastric tumors.** (A) Hierarchical clustering of the patterns of variation in expression of genes in 62 gastric tumors. Each row represents a separate cDNA clone on the microarray and each column represents the expression pattern in a separate tumor sample. The ratio of abundance of transcripts of each gene to its mean abundance across all tissue samples is depicted according to the color scale shown at the bottom. Gray indicates missing or excluded data. The dendrogram at the top of the figure represents the hierarchical clustering of the tumors based on similarity in their pattern of expression of these genes. (B) Clinical features of the 62 gastric tumors.
(PDF)

Figure S11 **Top biological functions of candidate genes identified by the ingenuity pathway analysis.**
(PDF)

Figure S12 **Compared ERBB2 expression values with the corresponding DNA copy number changes in 62 gastric cancer samples.**
(PDF)

Table S1 **Clinical parameters of 64 gastric cancer samples.**
(XLS)

Table S2 **The raw data of array-based CGH.**
(XLS)

Table S3 **DNA copy number variations in gastric cancer samples with different clinical parameters.**
(XLS)

Table S4 **Loci exhibiting high-level amplification or possible homozygous deletion.**
(PDF)

Table S5 **Genes show statistical significant correlation between expression values and DNA copy number variations.**
(XLS)

Table S6 **Candidate oncogenes and tumor suppressor genes identified by correlating expression arrays with aCGH data.**
(PDF)

Table S7 **Summary of analysis (IPA).**
(PDF)

Table S8 **The raw data of gene expression array.**
(XLS)
