# Supplementary material for: Integration of DNA Copy Number Alterations and Transcriptional Expression Analysis in Human Gastric Cancer
Source: PLoS One. 2012 Apr 23;7(4):e29824. doi: 10.1371/journal.pone.0029824 (PMC3335165; doi:10.1371/journal.pone.0029824)
Supplement: Figure S1 — Two representative genome-wide ratio plots for individual gastric tumor. Log2 ratio for each of the genomic clones was plotted according to chromosome position. (A) Whole genome DNA copy number profile of gastric cancer tissue sample HKG24T. Note that this sample showed the following DNA copy number variations: +3q, +5p, +8q, +13q, +17p, −4q, −10p and −18q. (B) Whole genome DNA copy number profile of gastric cancer cell line N87. Note that this sample showed the following DNA copy number variations: +5p, +8q, +11q, +20q, −3p, −5q, −6p, −6q, −7q, −8p, −11p, −14q, −17p and −21q. In addition, it also has amplification at 8q21, 8q24, 11q22 and 17q21. (PDF) [file pone.0029824.s001.pdf]

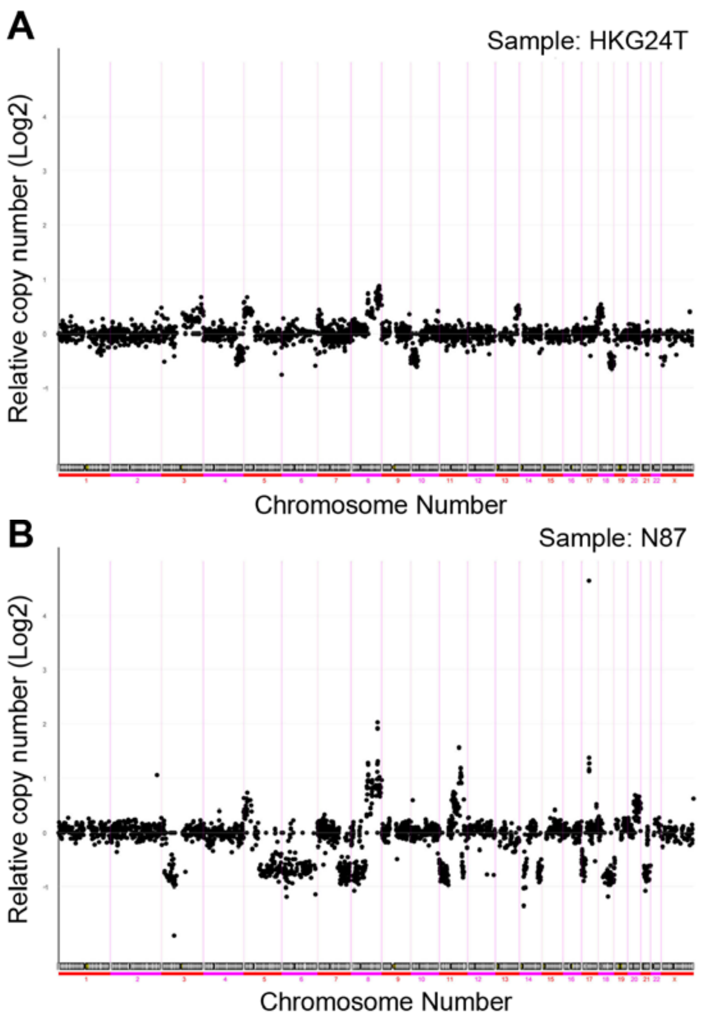

Figure S1. Two representative genome-wide ratio plots for individual gastric tumor. Log2 ratio for each of the genomic clones was plotted according to chromosome position. (A) Whole genome DNA copy number profile of gastric cancer tissue sample HKG24T. Note that this sample showed the following DNA copy number variations: +3q, +5p, +8q, +13q, +17p, -4q, -10p and -18q. (**B**) Whole genome DNA copy number profile of gastric cancer cell line N87. Note that this sample showed the following DNA copy number variations: +5p, +8q, +11q, +20q, -3p, -5q, -6p, -6q, -7q, -8p, -11p, -14q, -17p and -21q. In addition, it also has amplification at 8q21, 8q24, 11q22 and 17q21.
