# Supplementary material for: Integration of DNA Copy Number Alterations and Transcriptional Expression Analysis in Human Gastric Cancer
Source: PLoS One. 2012 Apr 23;7(4):e29824. doi: 10.1371/journal.pone.0029824 (PMC3335165; doi:10.1371/journal.pone.0029824)
Supplement: Figure S4 — DNA copy number variations in gastric cancer samples with different tumor stage. Data presented are ordered by chromosomal map position of the clones. Lower green bars represent losses or deletions, and the upper red bars represent gains or amplifications. (A) Stage 1 & 2 tumor. (B) Stage 3 & 4 tumor. (PDF) [file pone.0029824.s004.pdf]

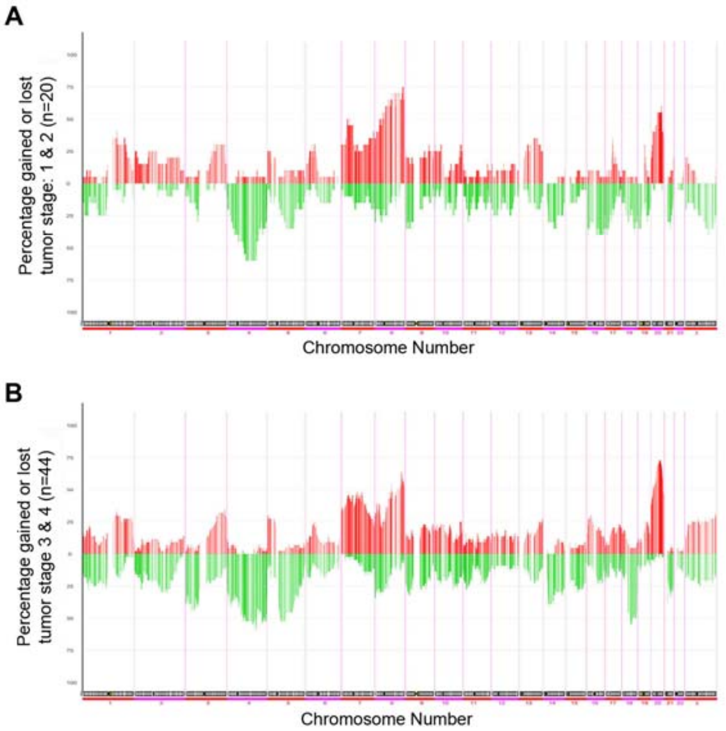

Figure S4. DNA copy number variations in gastric cancer samples with different tumor stage. Data presented are ordered by chromosomal map position of the clones. Lower green bars represent losses or deletions, and the upper red bars represent gains or amplifications. (A) Stage 1 & 2 tumor. (B) Stage 3 & 4 tumor.
