# Supplementary material for: Integration of DNA Copy Number Alterations and Transcriptional Expression Analysis in Human Gastric Cancer
Source: PLoS One. 2012 Apr 23;7(4):e29824. doi: 10.1371/journal.pone.0029824 (PMC3335165; doi:10.1371/journal.pone.0029824)
Supplement: Figure S5 — DNA copy number variations in gastric cancer samples with different tumor type. Data presented are ordered by chromosomal map position of the clones. Lower green bars represent losses or deletions, and the upper red bars represent gains or amplifications. (A) Tumor type: diffuse type. (B) Tumor type: intestinal type. (PDF) [file pone.0029824.s005.pdf]

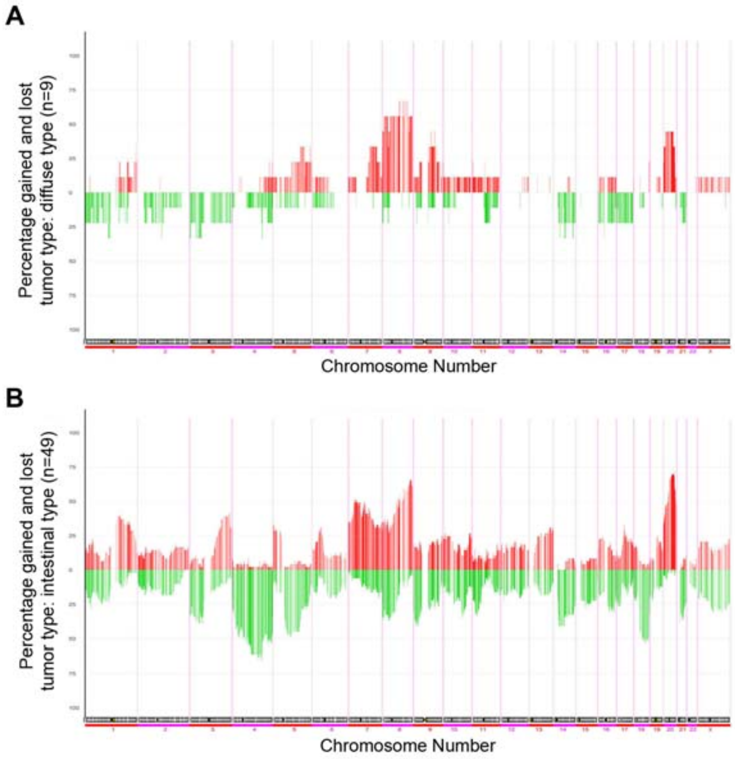

Figure S5. DNA copy number variations in gastric cancer samples with different tumor type. Data presented are ordered by chromosomal map position of the clones. Lower green bars represent losses or deletions, and the upper red bars represent gains or amplifications. (A) Tumor type: diffuse type. (B) Tumor type: intestinal type.
