# Supplementary material for: Integration of DNA Copy Number Alterations and Transcriptional Expression Analysis in Human Gastric Cancer
Source: PLoS One. 2012 Apr 23;7(4):e29824. doi: 10.1371/journal.pone.0029824 (PMC3335165; doi:10.1371/journal.pone.0029824)
Supplement: Figure S9 — Correlation between DNA copy number variations and global gene expression patterns. Each chromosomal arm was divided into equal number of parts or bins of size 20 Mb and then average pairwise Pearson correlation between gene expression and copy number was calculated for all pairs of binned regions. (A) Box plots of correlation between pairs along the diagonal (cDNA clones with surrounding BAC clones) and pairs off diagonal (cDNA clones with unrelated BAC clones). (B) Heatmap of the average correlation between gene expression and copy number. (PDF) [file pone.0029824.s009.pdf]

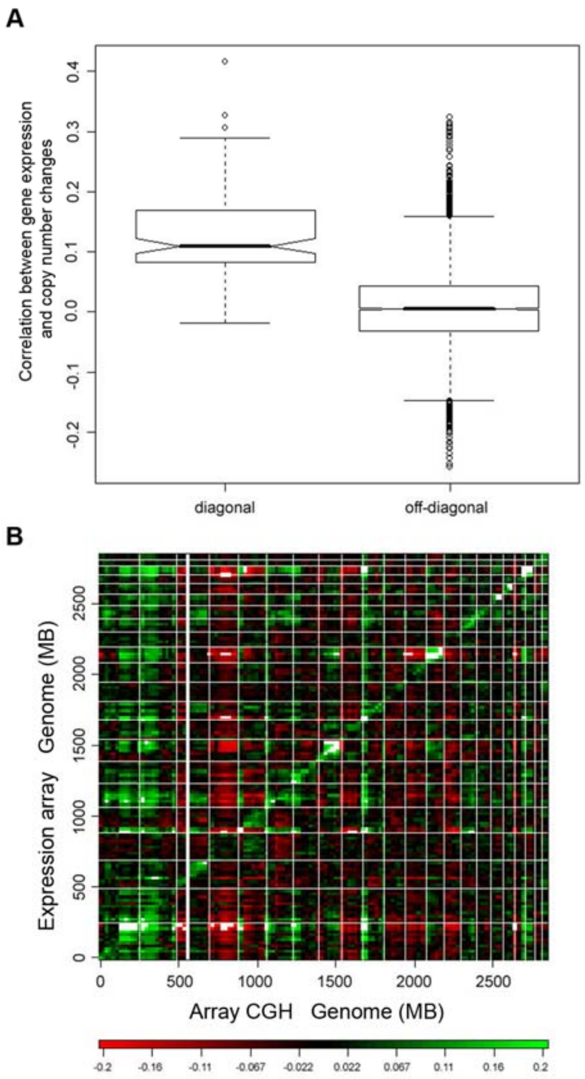

Figure S9. Correlation between DNA copy number variations and global gene expression patterns. Each chromosomal arm was divided into equal number of parts or bins of size 20Mb and then average pairwise Pearson correlation between gene expression and copy number was calculated for all pairs of binned regions. (A) Box plots of correlation between pairs along the diagonal (cDNA clones with surrounding BAC clones) and pairs off diagonal (cDNA clones with unrelated BAC clones). (B) Heatmap of the average correlation between gene expression and copy number.
