# Supplementary material for: Integration of DNA Copy Number Alterations and Transcriptional Expression Analysis in Human Gastric Cancer
Source: PLoS One. 2012 Apr 23;7(4):e29824. doi: 10.1371/journal.pone.0029824 (PMC3335165; doi:10.1371/journal.pone.0029824)
Supplement: Figure S10 — Correlations between the clustering pattern and clinical features of gastric tumors. (A) Hierarchical clustering of the patterns of variation in expression of genes in 62 gastric tumors. Each row represents a separate cDNA clone on the microarray and each column represents the expression pattern in a separate tumor sample. The ratio of abundance of transcripts of each gene to its mean abundance across all tissue samples is depicted according to the color scale shown at the bottom. Gray indicates missing or excluded data. The dendrogram at the top of the figure represents the hierarchical clustering of the tumors based on similarity in their pattern of expression of these genes. (B) Clinical features of the 62 gastric tumors. (PDF) [file pone.0029824.s010.pdf]

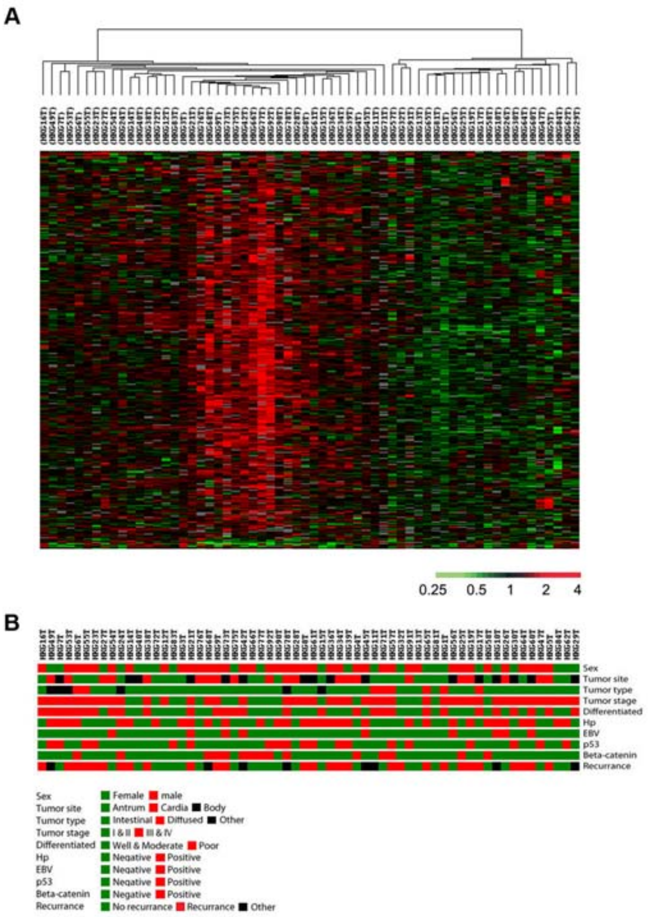


Figure S10. Correlations between the clustering pattern and clinical features of gastric tumors. (A) Hierarchical clustering of the patterns of variation in expression of genes in 62 gastric tumors. Each row represents a separate cDNA clone on the microarray and each column represents the expression pattern in a separate tumor sample. The ratio of abundance of transcripts of each gene to its mean abundance across all tissue samples is depicted according to the color scale shown at the bottom. Gray indicates missing or excluded data. The dendrogram at the top of the figure represents the hierarchical clustering of the tumors based on similarity in their pattern of expression of these genes. (B) Clinical features of the 62 gastric tumors.
