# Supplementary material for: Integration of DNA Copy Number Alterations and Transcriptional Expression Analysis in Human Gastric Cancer
Source: PLoS One. 2012 Apr 23;7(4):e29824. doi: 10.1371/journal.pone.0029824 (PMC3335165; doi:10.1371/journal.pone.0029824)
Supplement: Figure S11 — Top biological functions of candidate genes identified by the ingenuity pathway analysis. (PDF) [file pone.0029824.s011.pdf]

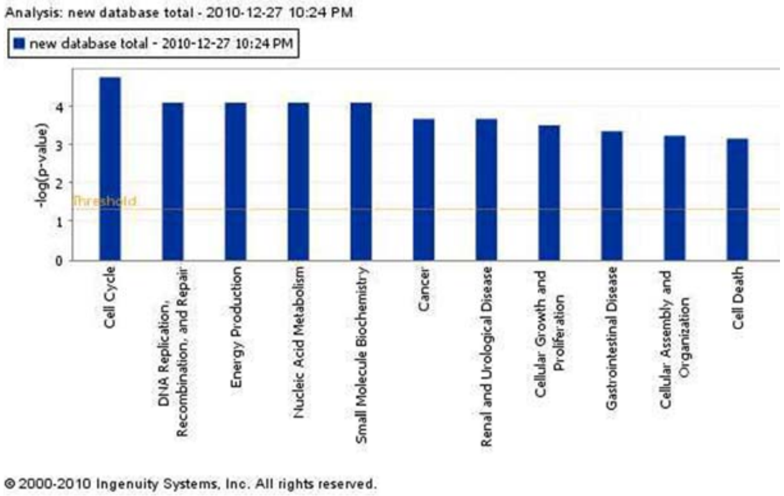

Figure S11. Top biological functions of candidate genes identified by the ingenuity pathway analysis.
