# Supplementary material for: Integration of DNA Copy Number Alterations and Transcriptional Expression Analysis in Human Gastric Cancer
Source: PLoS One. 2012 Apr 23;7(4):e29824. doi: 10.1371/journal.pone.0029824 (PMC3335165; doi:10.1371/journal.pone.0029824)
Supplement: Figure S12 — Compared ERBB2 expression values with the corresponding DNA copy number changes in 62 gastric cancer samples. (PDF) [file pone.0029824.s012.pdf]

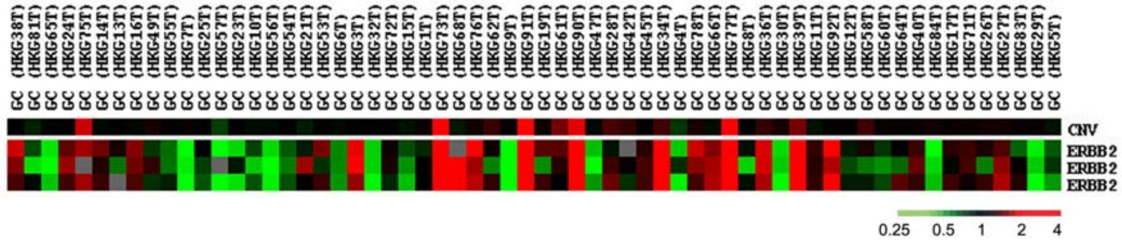

Figure S12. Compared ERBB2 expression values with the corresponding DNA copy number changes in 62 gastric cancer samples.
