# Supplementary material for: Integration of DNA Copy Number Alterations and Transcriptional Expression Analysis in Human Gastric Cancer
Source: PLoS One. 2012 Apr 23;7(4):e29824. doi: 10.1371/journal.pone.0029824 (PMC3335165; doi:10.1371/journal.pone.0029824)
Supplement: Table S4 — Loci exhibiting high-level amplification or possible homozygous deletion. (PDF) [file pone.0029824.s016.pdf]

Table S4. Loci exhibiting high-level amplification or possible homozygous deletion.

Alteration	Locus	Clone name	Frequency	Candidate gene
High-level amplication*	1q31	RP11-243M13	3/72	*SSA2, C1orf37, COX2, CNTN2, RBBP5, MDM4, LRRN5, jun, CYP2J*
	5p15.2	RP11-35K22, CTD-2040C2	5/72	*TRIO, CTNND2, DNAH5*
	5p15.3	RP11-94J21, RP11-58A5	5/72	
	5p14.3-5p15.1	RP11-88L18	3/72	*BASP1, CDH12, MLV12*
	5p13.2	RP11-253B9	3/72	*SKP2, SLC1A3, GDNF, IL7R, CAPSL, NUP155, WDR70, AMACR, RAD1*
	6p21.1	RP11-91H14	4/72	*CDC5L, TNF, Cip1, PIM1, PPP2R5D, CCND3, HGNC, MRLP14, HSPCB, NFKBIE, EGFL, RPL7L1, TRERF1, MRPL2, BYSL, MEA1, CCK-4, SRF, VEGF, PTK7, C6orf223, TMEM63B, CAPN11*
	7p12	RP11-183O1, RP11-15L23	4/72	*EGFR, ABCA13, ERBB1*
	7q21.12-7q21.13	CTB-102E19, CTB-141D22	3/72	*CDK6, AKAP9, CYP51A1, ABCB1, ABCB4, SRI, TP53AP1, PGY1, HGF*
	7q21.2-7q21.3	CTD-2074H8, RP11-127F3	5/72	*SMURF1, MCM7, PEG10*
	7q31.2-7q31.31	CTB-185C18	3/72	*MET, CFTR, Wnt2*
	8p22	RP11-112G9	3/72	*CTSB*
	8p23.1	RP11-235I5	3/72	*C8orf12, C8orf13, C8orf14, C8orf49, BLK, GATA4, NEIL2, MASL1, TNKS, MFHAS1,DEFA4, DLC1, CTSB*
	8q23.1	CTD-2013D21	3/72	
	8q24.1	RP11-128G18, RP11-145G10	7/72	*ZHX2, EXT1, ATAD2, NOV, OPG*

8q24.12-8q24.2	RP11-110D15	6/72	*ENPP2, ZHX2, MRPL13, MTBP, SNTB, SAMD12, TRIB1, RNF139, PRO2000, SQLE, KIAA0196, HAS2*
8q24.2	RP11-237F24, RP11-184M21	6/72	*MYC , PVT1, TG, DDEF1, SLA, MLZE, DDEF1, WISP1, NDRG1*
10q26.1	RP11-7P17	3/72	*KSAM/FGFR2*
11p12-11p13	RP11-90F13	5/72	*C11orf69, Q3C1V1, CD59, FBXO3, LMO2, CAPRIN1, LRRC4C, TRAF6, COMMD9, TRIM44, FJX1, CD44, PDHX, APIP, PKY/HIPK3, LMO2*
11p14	RP11-16H3, RP11-1L12	4/72	*GAS2*
11q21	RP11-163O18	3/72	*MAML2*
11q22.2	CTD-2039C11	3/72	*BIRC2, MMP1, MMP8, MMP10, MMP8, MMP13, YAP1, CASP4*
11q23.1	CTD-2222B22	3/72	*MLL, POU2AF1*
17q11.2-17q12		5/72	*TOP2A, GPR2, ERBB2/HER2,ZNF207, MLN 62, Pip4k2β, TRAF4 CPD, LASP1, SUPT6H, POLDIP2, FLOT2, PIGS, SARM1, PCFT, TIAF1, ERAL1, SEZ6, FLJ00234, TMEM97, TP53I13, CRYBA1, DHRS13, LOC201229, GIT1, NUFIP2, PIPOX, PHF12, ALDOC, RAB34, IFT20, UNC119, NLK, C17orf63, NEK8, SLC13A2, SDF2, FLJ00295, PROCA1, DKFZp547N203, FOXN1, TLCD1, VTN, RPL23A, SPAG5, FLJ40504, LOC116236, MYO18A, UNQ419, BCOX, TNFAIP1, TAOK1, SEBOX, C17orf32, ACCN1, MLLT6*

17q12-17q21	RP11-87N6	3/72	*PPP1R1B/DARPP32, RAPGEFL1, MLN 64/ TRAF4/ STARD3, MLN50, PPARBP/ TRAP220, GRB7, PEX12, NEUROD2, PERLD1, PLXDC1, CACNB1, RPL19, HSD17B1, THRA/ERBA1, RARA, MLN51, IGFBP4, WT4, CCR7, CDC6, ZNF144, CCL8, CCL1, CCL2, CCL11, CCL7, CCL13, AATF, TBC1D3*
17q21.1-17q21.2	CTD2009O18, CTD-2094C6	5/72	*Keratin familys, GAST*
17q21-17q22	GS-265E19	3/72	*SGCA, HOXB7, FLJ13855, EAP30, PHB, PPP1R9B, FHF3/RHO7, NGFR, SHCL1, NME1/NM23, NME2, GIP, ZNF147, ABCC3, RPS6KB1, ABC1, KIF2B, GOSR2, NDP52, TOM1L1, COX11, ITGA3, PPP1R9B, PDK2, MYST2, COL1A1, FAM117A, TAC4, DLX3, TMEM92, DLX4, SAMD14, SGCA, HILS1, FLJ42842, CA10*
19p11-19q11	RP11-206O6, RP11-110J19	6/72	
19q11-19q12	RP11-152P7, CTB-25O22	8/72	*VCC-1, UQCRFS1,*
19q12-19q13.1	RP11-46I12	6/72	*CCNE1, C19orf12, HPN/HEPSIN, SPINT2, Cyclin E/CCNE, CEBPA, TGFB1, BCL3, OZF, SEI-1, MLL2, ACTN4, PD2/hPaf1, AKT2, Mirk/ Dyrk1B*
20q12	RP11-93L19, RP11-30F23	7/72	*AIB1/ ACTR/ RAC3/ TRAM-1/ p/CIP/ SRC-3,*
20q12-20q13.1	RP11-169A6, CTD-2031K20	12/72	*SLPI, HE4, TOP1, CAS, MYBL2*

	20q13.1-20q13.2	GS-236D3, RP11-172C21	11/72	*C20orf17, ZNF217, BCAS1, PTPN1, LOC284751, KIAA0611, ATP9A, BCAS1, CYP24, PFDN4, CSE1L, NCOA3, BMP7, RAB22A, STK4, C20orf121, BCAS4, SULF2, EYA2*
	20q13.2-20q13.3	GS-32I19, RP11-1E23	6/72	*BTAK/STK15/STK6, TFAP2C, NABC1, GNAS, ADRM1, AURKA*
	Xp22.1	RP11-48D14, CTD-2033A1	3/72	
	Xp22.2	RP11-2K15, RP11-128B23	4/72	
	Xp22.3	RP4-677N1	3/72	
Homozygous deletion[#]	3p14	RP11-94D19	5/72	*PTPRG, PTPγ,*
	3p14.2	CTB-1O12	5/72	*FHIT*
	3p14.3	CTD-2175D15	3/72	*WNT5A, TU3A*
	4p12-13	RP11-24G16	3/72	*PHOX2B, LOC401131, HUEL, UGT2B17*
	4p16.2	RP11-7A6	3/72	*DOK7*
	4q22	RP11-49M7	3/72	*UNC5C, GRID2, DRLM, LIM, ALK6, RAP1, DAPP1, MP1, TMSL3*
	4q28	RP11-40D5	3/72	
	5p14.3-15.1	RP11-88L18	4/72	*CDH6, CDH10, NKD2, CDH18*
	5q11.2	RP11-22L20	3/72	*MEKK1, BACTIL, hMSH3, PDE4D, MAP3K1, LOC54505, GZMK, GZMA, CKN1*
	5q12	RP11-19I19	5/72	*LY64, PART-1*
	5q13	CTB-134O19	3/72	*F2RL1, F2R, RAD17, PIK3R1*
	8p12	RP11-57I3, RP11-210F15	3/72	*VDAC3, sFRP1, FRP1/FRZB, WRN, NRG1*
	8p21.2	RP11-76B12	3/72	*FEZ, DR5, NKX3, CLU, TNFRSF10B, KAI1, NKX3.1,*

			NEFL, LPL, BNIP3L, PPP2R2A, DOCK5, RHOBTB2
8p23.1	RP11-122N11	3/72	*DEFB105A, DEFB106A, LPTS, DLC1, PinX1, CSMD1, GATA4, FDFT1, NEIL2*
8p23.3	RP11-82K8	3/72	*DLGAP2, FBOXO25, ARHGEF10, KBTBD11*
8ptel	GS1-77L23	4/72	
9p21	CTB-65D18, RP11-235F7	8/72	*p16/ INK4A/CDKN2A, p14ARF, p15/CDKN2B, 189G21T7, D9S171, TLE1, PTENP1, IFNA, MTAP, TEK, MLLT3*
11p15.1	RP11-56J22	3/72	*KIP2, BGLOB, WEE, TSG101, CDKN1C /p57KIP2*
16q23	RP11-61L1, RP11-284G2	5/72	*FOR/WWOX/WOX, WI2755/FRA16D, ADAMTS18, TERF21P, ATBF1*
18q12	RP11-104N11	5/72	
18q12-21	RP11-19L3	3/72	*NPM1P1, PIK3C3, RIT2SYT4, SETBP1, SLC14A2, SLC14A1, CD33L3, KIAA1632, PSTPIP2*
18q21	RP11-43K24, RP11-729G3	4/72	*OPC4, MBD2, ME2, ELAC1, CDH20, MALT1, GRP, FVT1, SERPINB5/PI5, SERPINB13, SERPINB4, SERPINB3*
18q21.1	VYSIS-DPC4?	3/72	*MADH4/DPC4/SMAD4, MADR2 /SMAD2/JV18-1, DCC, MADH7/SMAD7, LOC390855*
18q22	RP11-748M14, RP11-105L16	6/72	*CDH19, CADH-7, BCL2, DOK6, CD226, RTTN, CBLN2, NETO1, TMX3, CCDC102B*
18q22-18q23	RP11-49H23, RP11-57F7	4/72	*DNAM-1, CTDP1*
18q23	RP11-64C15, RP11-130J1	5/72	*SOCS6, GALR1, MBP*
18qtel	RP11-507P3	3/72	*CTDP1*

21q11.1-21q11.2	RP11-31B6	4/72	*LIPI, RBM11, STCH, SAMSN1, NRIP1, USP25, C21orf34, CXADR, BTG3/ANA, C21orf91, CHODL, PRSS7, JAM2*
21q21	RP11-15H6	3/72	*ANA, ADAMTS5, ADAMTS1, BTG3, MIRN99A, MIRN125B2, MIRNLET7C, USP25, YG81, BIC*
21q22.2-21q22.3	RP11-1P3	3/72	*TMPRSS2, COL6A1, DSCAM, BACE2, PLAC4*

* Log2 > 0.8. # Log2 < -0.7.
