# Supplementary material for: Integration of DNA Copy Number Alterations and Transcriptional Expression Analysis in Human Gastric Cancer
Source: PLoS One. 2012 Apr 23;7(4):e29824. doi: 10.1371/journal.pone.0029824 (PMC3335165; doi:10.1371/journal.pone.0029824)
Supplement: Table S6 — Candidate oncogenes and tumor suppressor genes identified by correlating expression arrays with aCGH data. (PDF) [file pone.0029824.s018.pdf]

Table S6. Candidate oncogenes and tumor suppressor genes identified by correlating expression arrays with aCGH data.

Genes that are up-regulated and are frequently gained in human GCs							
Gene Symbol	Chr	Percentage Gained*	Fold Up Regulation#	Gene Symbol	Chr	Percentage Gained*	Fold Up Regulation#
RNF115	1	17.74	1.41	ESRP1	8	17.74	1.41
BCL9	1	17.74	1.33	PTDSS1	8	19.35	1.52
SF3B4	1	20.97	1.6	MTDH	8	19.35	2.06
ANP32E	1	20.97	2.22	LAPTM4B	8	19.35	1.55
TARS2	1	20.97	1.52	STK3	8	19.35	1.82
PRUNE	1	20.97	1.36	COX6C	8	19.35	1.38
S100A7	1	20.97	1.51	AZIN1	8	24.19	1.65
ILF2	1	20.97	1.69	SLC25A32	8	24.19	1.56
SLC39A1	1	20.97	1.62	NUDCD1	8	24.19	2.08
TPM3	1	20.97	1.52	RAD21	8	25.81	1.8
UBE2Q1	1	20.97	1.28	DSCC1	8	25.81	1.56
FLAD1	1	20.97	1.6	MRPL13	8	25.81	1.83
EFNA1	1	20.97	1.37	DERL1	8	30.65	1.27
GBA	1	20.97	1.29	ATAD2	8	30.65	2.58
FAM189B	1	20.97	1.39	FAM91A1	8	30.65	2.6
ASH1L	1	20.97	1.51	PVT1	8	40.32	2.11
MSTO1	1	20.97	1.33	FAM49B	8	37.1	1.92
DAP3	1	20.97	1.6	ASAP1	8	37.1	1.85
SMG5	1	20.97	1.41	PHF20L1	8	37.1	1.43
GPATCH4	1	20.97	1.37	EIF2C2	8	35.48	1.37
MRPL24	1	20.97	1.31	PTK2	8	37.1	1.52
NUF2	1	20.97	2.17	RHPN1	8	35.48	1.54
UCK2	1	20.97	1.53	PUF60	8	35.48	1.6
CREG1	1	22.58	1.88	GRINA	8	35.48	2.22
CACYBP	1	19.35	2.1	EXOSC4	8	35.48	1.29
STX6	1	17.74	1.44	C8orf30A	8	35.48	1.44
SMG7	1	19.35	1.31	FBXL6	8	35.48	1.58
ARPC5	1	19.35	1.6	RECQL4	8	35.48	2.07
TPR	1	19.35	1.47	ARHGAP39	8	35.48	1.78
ZNF281	1	16.13	1.75	C8orf33	8	35.48	1.65
KIF21B	1	16.13	1.39	SETX	9	16.13	1.44
IPO9	1	16.13	1.62	NOTCH1	9	20.97	2.41
ARL8A	1	16.13	1.3	GTPBP4	10	11.29	1.97
UBE2T	1	14.52	2.06	PFKP	10	12.9	1.4
KDM5B	1	14.52	1.3	TAF3	10	11.29	1.31
SNRPE	1	14.52	1.82	UPF2	10	12.9	1.51
NUCKS1	1	14.52	1.46	NUDT5	10	12.9	1.46
CD46	1	16.13	1.85	CDC123	10	12.9	1.52
TRAF5	1	16.13	1.51	SEPHS1	10	11.29	1.49
NEK2	1	14.52	1.71	CD9	10	11.29	1.59
SMYD2	1	14.52	1.59	MLLT10	10	9.68	1.35

CENPF	1	14.52	2.83
LYPLAL1	1	12.9	1.47
IARS2	1	12.9	2.14
TAF1A	1	12.9	1.62
CNIH4	1	16.13	1.34
LBR	1	14.52	2.19
LIN9	1	14.52	2.08
PARP1	1	14.52	1.8
ABCB10	1	14.52	1.46
C1orf198	1	14.52	2.07
TSNAX	1	14.52	1.66
C1orf31	1	12.9	1.42
TARBP1	1	12.9	1.48
IRF2BP2	1	12.9	1.68
CHML	1	12.9	1.54
ADSS	1	11.29	1.66
FAM36A	1	11.29	1.74
AHCTF1	1	11.29	1.93
LAPTM4A	2	12.9	1.38
DHX57	2	14.52	1.46
VPS54	2	8.06	1.33
RUVBL1	3	9.68	2.07
RAB7A	3	11.29	1.43
CDV3	3	9.68	1.39
ATP1B3	3	12.9	2.36
HPS3	3	16.13	1.71
CP	3	16.13	1.47
GFM1	3	16.13	1.6
ECT2	3	17.74	3.66
ACTL6A	3	17.74	2.03
FXR1	3	17.74	1.84
DVL3	3	19.35	1.49
ECE2	3	19.35	1.78
EIF4G1	3	19.35	1.48
CLCN2	3	19.35	1.49
MAGEF1	3	19.35	1.32
RFC4	3	19.35	2.03
PPP1R2	3	17.74	1.27
FBXO45	3	17.74	1.83
SENP5	3	17.74	1.77
CCDC127	5	29.03	1.26
PDCD6	5	29.03	1.36
TRIP13	5	29.03	1.83
NKD2	5	29.03	1.53
SLC12A7	5	29.03	1.33
LPCAT1	5	29.03	2.17
BMI1	10	11.29	1.8
WAC	10	11.29	1.4
MAP3K8	10	11.29	1.59
KIF5B	10	11.29	1.56
PARD3	10	12.9	1.35
METTL10	10	12.9	1.46
BCCIP	10	12.9	1.58
GLRX3	10	11.29	1.61
DYRK2	12	8.06	1.31
RAP1B	12	8.06	1.33
NUP107	12	8.06	2
MDM2	12	8.06	1.69
CPSF6	12	9.68	1.72
FRS2	12	9.68	1.27
RAB3IP	12	9.68	1.49
POMP	13	14.52	1.59
SLC7A1	13	14.52	1.45
RFC3	13	11.29	2.03
C13orf23	13	11.29	1.52
DGKH	13	11.29	1.95
NUFIP1	13	11.29	1.54
ZC3H13	13	11.29	1.49
RNASEH2B	13	17.74	1.55
PIBF1	13	12.9	1.75
TGDS	13	14.52	1.49
RAP2A	13	14.52	1.65
IPO5	13	14.52	1.64
DOCK9	13	14.52	1.93
ING1	13	17.74	1.49
ANKRD10	13	17.74	1.99
ATP11A	13	19.35	1.92
CUL4A	13	19.35	1.51
TFDP1	13	14.52	1.79
MED1	17	17.74	1.57
CDK12	17	17.74	1.56
GRB7	17	17.74	1.44
PSMD3	17	12.9	1.59
CDC6	17	12.9	1.99
TOP2A	17	12.9	2.27
KRT23	17	11.29	1.88
ACLY	17	14.52	1.64
EFTUD2	17	11.29	1.33
PHB	17	11.29	1.33
MED13	17	12.9	1.51
TANC2	17	12.9	1.36
PRKCA	17	11.29	1.33

MED10	5	22.58	1.27
NSUN2	5	22.58	1.82
PAPD7	5	22.58	1.34
FAM173B	5	22.58	1.34
CCT5	5	22.58	1.89
TRIO	5	25.81	1.62
ANKH	5	22.58	1.87
RNASEN	5	22.58	1.59
ZFR	5	27.42	1.82
SUB1	5	27.42	1.48
RAD1	5	25.81	1.6
BRIX1	5	25.81	2.33
C5orf33	5	25.81	1.4
NIPBL	5	24.19	1.45
C5orf28	5	24.19	1.31
MRPS30	5	24.19	1.48
FLOT1	6	14.52	1.3
TCF19	6	14.52	1.67
CSNK2B	6	14.52	1.3
MSH5	6	14.52	1.29
VARS	6	14.52	1.3
EHMT2	6	14.52	1.3
SNRPC	6	16.13	1.48
TOMM6	6	19.35	1.48
BYSL	6	19.35	1.73
RPL7L1	6	19.35	1.29
MEA1	6	19.35	1.27
CUL7	6	19.35	1.26
XPO5	6	20.97	1.92
CDC5L	6	17.74	1.43
INTS1	7	12.9	1.37
EIF3B	7	11.29	1.73
C1GALT1	7	11.29	1.53
C1GALT1	7	11.29	1.34
MACC1	7	11.29	1.54
KLHL7	7	11.29	1.69
HNRNPA2B1	7	12.9	1.93
CBX3	7	12.9	1.62
SKAP2	7	12.9	1.65
LOC100289444	7	12.9	1.89
HOXA13	7	12.9	1.65
AVL9	7	12.9	1.82
KBTBD2	7	11.29	1.75
FKBP9	7	11.29	1.44
STARD3NL	7	11.29	1.6
PSMA2	7	8.06	1.43
NOL11	17	11.29	1.71
FDXR	17	11.29	1.51
SUMO2	17	11.29	1.38
RBCK1	20	27.42	1.45
TBC1D20	20	27.42	1.28
SIRPA	20	27.42	1.84
STK35	20	27.42	1.33
SNRPB	20	27.42	1.39
MRPS26	20	27.42	1.49
CDC25B	20	29.03	1.62
PCNA	20	25.81	1.75
GPCPD1	20	24.19	1.53
PLCB1	20	27.42	1.34
MKKS	20	27.42	1.34
ESF1	20	30.65	2.2
SNX5	20	29.03	1.49
NAA20	20	30.65	1.53
NANP	20	35.48	1.58
HM13	20	41.94	1.39
TPX2	20	41.94	3.2
TM9SF4	20	38.71	1.27
PLAGL2	20	32.26	1.59
POFUT1	20	32.26	1.88
E2F1	20	41.94	1.48
AHCY	20	41.94	1.39
ITCH	20	41.94	1.45
PIGU	20	41.94	1.35
PROCR	20	41.94	1.34
CPNE1	20	41.94	1.88
NFS1	20	41.94	1.54
RBM39	20	41.94	1.41
DLGAP4	20	41.94	1.3
C20orf24	20	41.94	1.46
C20orf117	20	41.94	1.39
RPN2	20	41.94	1.72
BLCAP	20	40.32	1.67
RPRD1B	20	40.32	1.64
IFT52	20	41.94	1.61
MYBL2	20	41.94	2.17
C20orf111	20	41.94	1.4
SERINC3	20	41.94	1.68
YWHAB	20	41.94	1.36
TOMM34	20	41.94	1.36
STK4	20	41.94	1.71
UBE2C	20	41.94	2.97
TP53RK	20	41.94	1.25

C7orf44	7	8.06	1.35	NCOA3	20	41.94	1.68
H2AFV	7	8.06	1.35	ZNFX1	20	41.94	1.62
PURB	7	8.06	1.47	NCRNA00275	20	41.94	1.75
IGFBP3	7	8.06	2.62	RNF114	20	43.55	1.66
TNS3	7	8.06	1.39	DPM1	20	41.94	1.61
MRPS17	7	11.29	1.6	ATP9A	20	41.94	1.31
SLC25A40	7	8.06	1.79	ZNF217	20	40.32	1.51
SRI	7	8.06	1.27	AURKA	20	37.1	2.36
FZD1	7	8.06	1.27	C20orf43	20	37.1	1.61
ANKIB1	7	8.06	1.48	RAE1	20	37.1	1.72
PEX1	7	8.06	1.27	RBM38	20	37.1	1.47
CDK6	7	8.06	1.55	PMEPA1	20	37.1	3.05
SAMD9L	7	8.06	1.49	RAB22A	20	37.1	1.65
CCDC132	7	8.06	1.51	VAPB	20	37.1	1.37
MCM4	8	16.13	2.14	VAPB	20	37.1	1.32
UBE2V2	8	16.13	1.56	STX16	20	37.1	1.79
CHCHD7	8	16.13	1.27	GNAS	20	37.1	1.32
IMPAD1	8	16.13	1.78	ATP5E	20	37.1	1.22
SNHG6	8	17.74	1.59	CDH26	20	35.48	1.8
CSPP1	8	17.74	1.57	PSMA7	20	30.65	1.66
NCOA2	8	22.58	1.31	LAMA5	20	30.65	1.78
TRAM1	8	22.58	1.47	COL9A3	20	30.65	2.01
LACTB2	8	22.58	2.06	C20orf11	20	30.65	1.4
MRPS28	8	17.74	1.4	ARFGAP1	20	30.65	1.27
IMPA1	8	17.74	1.78	SLC2A4RG	20	33.87	1.36
OSGIN2	8	17.74	2.22	TPD52L2	20	35.48	1.75
NBN	8	17.74	1.96	PRPF6	20	35.48	1.31
PDP1	8	17.74	1.83				

Genes that are down-regulated and are frequently lost in human GCs

Gene Symbol	Chr	Percentage Lost*	Fold Down Regulation#	Gene Symbol	Chr	Percentage Lost*	Fold Down Regulation#
SYTL1	1	8.06	1.75	UQCRQ	5	9.68	1.52
FAM107A	3	12.9	2.27	MYO5B	18	24.19	1.58
S100P	4	12.9	3.48	NEDD4L	18	24.19	1.58
OCIAD2	4	9.68	1.37	SEC11C	18	24.19	1.6
PLAC8	4	9.68	3.27	CYB5A	18	25.81	2.1
IQGAP2	5	17.74	1.48	CBR1	21	11.29	2.25

*Percentage of GC samples with gain or loss at the specific gene loci. #Average fold of up-regulation or down-regulation of the gene comparing GC with non-tumor gastric samples.
