# Supplementary material for: Integration of DNA Copy Number Alterations and Transcriptional Expression Analysis in Human Gastric Cancer
Source: PLoS One. 2012 Apr 23;7(4):e29824. doi: 10.1371/journal.pone.0029824 (PMC3335165; doi:10.1371/journal.pone.0029824)
Supplement: Table S7 — Summary of analysis (IPA). (PDF) [file pone.0029824.s019.pdf]

IPA® Summary of Analysis

Analysis Name: new database total - 2010-12-27 10:24 PM
Analysis Creation Date: 2010-12-27
IPA version: 8.8 (Release Date: 2010-11-13)
Content version: 3204 (Release Date: 2010-10-27)

Analysis settings

View

Reference set: Ingenuity Knowledge Base (Genes + Endogenous Chemicals)

Relationship to include: Direct and Indirect

Includes Endogenous Chemicals

Optional Analyses: My Pathways My List

Filter Summary:

Consider only molecules and/or relationships where

species = Human

Top Networks

ID	Associated Network Functions	Score
1	Cancer, Renal and Urological Disease, Cell Cycle	29
2	Connective Tissue Development and Function, Cancer, Gastrointestinal Disease	26
3	Cell Cycle, Endocrine System Development and Function, Small Molecule Biochemistry	21
4	Genetic Disorder, Skeletal and Muscular Disorders, Inflammatory Disease	2

(c) 2000-2010 Ingenuity Systems, Inc. All rights reserved.

(c) 2000-2010 Ingenuity Systems, Inc. All rights reserved.

Top Bio Functions

Diseases and Disorders

Name	p-value	# Molecules
Cancer	2.21E-04 - 4.93E-02	18
Renal and Urological Disease	2.21E-04 - 2.98E-02	3
Gastrointestinal Disease	4.61E-04 - 3.07E-02	10
Genetic Disorder	9.47E-04 - 4.13E-02	26
Connective Tissue Disorders	1.38E-03 - 7.54E-03	2

Molecular and Cellular Functions

Name	p-value	# Molecules
Cell Cycle	1.90E-05 - 4.93E-02	12
DNA Replication, Recombination, and Repair	8.34E-05 - 4.44E-02	12
Energy Production	8.34E-05 - 8.34E-05	3
Nucleic Acid Metabolism	8.34E-05 - 8.34E-05	3
Small Molecule Biochemistry	8.34E-05 - 1.75E-02	4

Physiological System Development and Function

Name	p-value	# Molecules
Hematological System Development and Function	9.47E-04 - 3.47E-02	4
Hematopoiesis	9.47E-04 - 3.47E-02	3
Connective Tissue Development and Function	2.38E-03 - 4.93E-02	8
Embryonic Development	2.52E-03 - 2.00E-02	4
Nervous System Development and Function	2.52E-03 - 4.93E-02	2

(c) 2000-2010 Ingenuity Systems, Inc. All rights reserved.

INGENUITY SYSTEMS

Top Canonical Pathways

Name	p-value	Ratio
Mismatch Repair in Eukaryotes	7.25E-04	2/20 (0.1)
Role of CHK Proteins in Cell Cycle Checkpoint Control	3.11E-03	2/35 (0.057)
Role of BRCA1 in DNA Damage Response	8.73E-03	2/58 (0.034)
Valine, Leucine and Isoleucine Biosynthesis	2.98E-02	1/12 (0.083)
Hereditary Breast Cancer Signaling	3.44E-02	2/122 (0.016)

Top Molecules

Fold Change up-regulated

Molecules	Exp. Value	Exp. Chart
ECT2	↑1.870	
TPX2	↑1.680	
PMEPA1	↑1.610	
UBE2C	↑1.570	
CENPF	↑1.500	
FAM91A1	↑1.380	
ATAD2	↑1.370	
NOTCH1	↑1.270	
ATP1B3	↑1.240	
AURKA	↑1.240	

Fold Change down-regulated

Molecules	Exp. Value	Exp. Chart
S100P	↓-1.800	
PLAC8	↓-1.710	

(c) 2000-2010 Ingenuity Systems, Inc. All rights reserved.

FAM107A	↓-1.180
CBR1	↓-1.170
CYB5A	↓-1.070

(c) 2000-2010 Ingenuity Systems, Inc. All rights reserved.

Top My Lists

Name	p-value	Ratio

Top My Pathways

Name	p-value	Ratio

Top Tox Lists

Name	p-value	Ratio
Cell Cycle: G2/M DNA Damage Checkpoint Regulation	8.23E-02	1/34 (0.029)
Hypoxia-Inducible Factor Signaling	1.62E-01	1/70 (0.014)
NRF2-mediated Oxidative Stress Response	3.8E-01	1/188 (0.005)

(c) 2000-2010 Ingenuity Systems, Inc. All rights reserved.

INGENUITY® SYSTEMS

Top Tox Functions

Assays: Clinical Chemistry and Hematology

Name	p-value	# Molecules
Increased Levels of Alkaline Phosphatase	9.61E-02 - 9.61E-02	1

Cardiotoxicity

Name	p-value	# Molecules
Cardiac Damage	3.23E-02 - 3.23E-02	1
Congenital Heart Anomaly	5.40E-02 - 5.40E-02	1
Cardiac Arteriopathy	1.00E00 - 1.00E00	2

Hepatotoxicity

Name	p-value	# Molecules
Liver Cirrhosis	4.96E-02 - 1.75E-01	2
Hepatocellular Carcinoma	1.01E-01 - 1.01E-01	2

Nephrotoxicity

Name	p-value	# Molecules
Renal Transformation	1.00E-02 - 1.00E-02	1

(c) 2000-2010 Ingenuity Systems, Inc. All rights reserved.
